# Systematic curation of protein and genetic interaction data for computable biology

**DOI:** 10.1186/1741-7007-11-43

**Published:** 2013-04-15

**Authors:** Kara Dolinski, Andrew Chatr-aryamontri, Mike Tyers

**Affiliations:** 1 Lewis-Sigler Institute for Integrative Genomics, Princeton University, Princeton, NJ 08544, USA; 2 Institute for Research in Immunology and Cancer, Université de Montréal, Montréal, H3C 3J7, Canada

## 

Research over the past three decades has revealed that cellular behavior is governed by dynamic, complex networks of interactions among proteins, RNA, DNA, lipids and metabolites [1]. As such, each discrete interaction represents the minimal input unit for computational models of biological system responses. This principle in turn requires that biological interactions be rigorously documented in a readily computable format to enable mathematical models of network function that are able to predict non-obvious behavior. Despite the foundational shifts in our conception of biology, however, we still use the same primary method to disseminate scientific information that was used by Darwin over 150 years ago, namely the free-text, descriptive narrative style of conventional publications. The vast and ever-increasing biomedical literature thus poses a formidable challenge for the annotation of biological data and computational analysis [2].

Our 2006 publication describing the comprehensive curation of protein and genetic interactions for the budding yeast *Saccharomyces cerevisiae *began to address this data challenge by completing the first systematic effort to convert the interaction data embedded in the biomedical literature into a computable format [3]. This curated dataset allowed biomedical researchers to rapidly query, visualize, and analyze the entirety of the yeast literature for biological interactions. The dissemination and interrogation of the interaction dataset was facilitated by development of an open access database called BioGRID and an associated graphical viewer called Osprey. Initially, our original data set served primarily as a benchmark for high-throughput (HTP) protein interaction datasets that we and others had generated, and as a look-up table for hypothesis generation by individual researchers [3]. The BioGRID now contains more than 500,000 interactions across some 30 different model organism species [4]. Comprehensive literature curation has also been completed for the fission yeast *Schizosaccharomyces pombe *and the thale cress *Arabidopsis thaliana*. In parallel, curation projects on human interactions in themed areas of biomedical interest have been undertaken. These datasets are disseminated by many different partner databases and meta-resources, including the *Saccharomyces *Genome Database [5] and other model organism databases, the Gene Ontology Consortium [6], the International Molecular Exchange (IMEx) Consortium [7], and the Pathway Commons initiative [8]. The Proteomics Standards Initiative-Molecular Interactions (PSI-MI) standard has been developed by an international consortium to unify experimental evidence codes for protein interactions across databases [7], and analogous standards are now being developed by BioGRID and its partners for genetic interactions and quantitative phenotypic traits. The BioGRID dataset has been kept current with the literature through archived monthly updates, and has found numerous applications, from the analysis of biological network properties, to predictions of gene function, to the interpretation of genetic interactions, to a standard for automated text mining approaches [4].

Since our original publication, the rate of generation of molecular interaction data has exploded. Over the past several years, a variety of different HTP approaches have been used to chart protein and genetic interaction networks, which have greatly extended the scope of biological interaction space and motivated many hypothesis-driven studies. In yeast, the protein interaction landscape has grown from about 23,000 non-redundant interactions in 2006 to over 75,000 at present, while the number of genetic interactions measured by synthetic growth effects has increased from about 14,000 non-redundant interactions in 2006 to over 140,000 at present [3,4]. HTP methods for detection of protein and nucleic acid interactions have also enabled the comprehensive inference of regulatory relationships [9]. A host of analogous systematic detection approaches have now begun to chart the extensive networks of interactions and associated protein modifications in human cells [10]. Even in yeast, however, the full extent of the protein and genetic interactomes, and the regulatory relationships that connect the two, remain to be determined.

The availability of robust datasets derived from the primary literature and HTP studies has enabled graphical representation and interrogation of global interaction networks, and the prediction of gene and network function [11]. Such tools are essential for de-convolution of the now commonplace but inscrutable interaction 'hairball' (Figure 1), which belies the regulatory logic that is encoded in complex networks [1]. These methods have begun to allow analysis of networks implicated in human disease and the identification of critical nodes as therapeutic targets [10]. This network approach to understanding disease should not only identify new targets for drug discovery but should also predict drug combinations tailored to compensate for specific network mutations [12].

**Figure 1:**
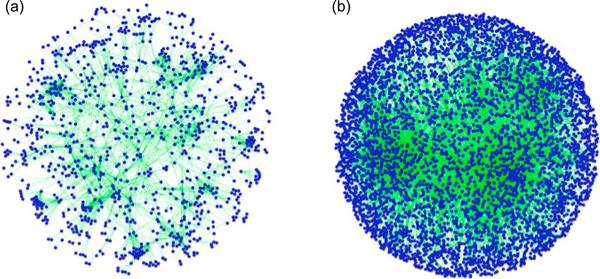
**Growth of the interaction hairball**. **(a) **Network graph representation of the BioGRID interaction dataset in 2006 representing 157,123 genetic and physical interactions. **(b) **Network graph representation of the BioGRID interaction dataset in 2013 representing 638,453 genetic and physical interactions. Both graphs are scaled down to 1/30^th ^of actual size to simplify the representation. Datasets were drawn from BioGRID releases 2.0.18 for 2006 and 3.2.97 for 2013. Graphical representations were built using the Cytoscape visualization platform.

Despite the striking experimental progress that has ushered in the era of the hairball [1], the annotation and computational analysis of interaction datasets is still at a nascent stage. A fundamental issue with expert manual curation is the rate of growth of the primary literature, which manifestly outstrips the rate of curation (Figure 2). To put this problem in perspective, PubMed currently contains over 22,000,000 publication entries (some 12,000,000 of which pertain to human biology), and new publications are accumulating at a rate of approximately two every minute. Although automated text-mining approaches can expedite curation, these approaches are inherently limited by the inadequacies of natural language processing algorithms, and it is clear that much of the literature will remain opaque to computation unless experimental interaction data are explicitly annotated as a part of the publication process. A simple and cost-effective solution would be to mandate the deposition of structured records that rigorously describe experimental evidence and quantitative parameters for biological interactions as an inherent part of the publication process.

**Figure 2:**
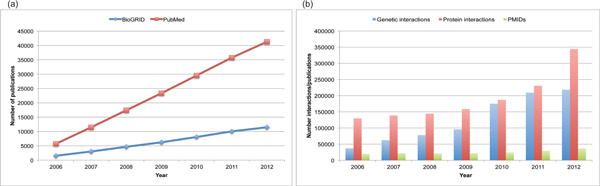
**The inflationary publication problem**. **(a) **Accumulation of total protein and genetic interactions for all species in BioGRID over time. The number of publications from which the interactions are derived is also shown. **(b) **Increase in publications likely to contain interaction data in PubMed over time versus actual publications curated for interactions across all species in BioGRID. Estimation of PubMed entries likely to contain interaction data was performed using the Protein Interaction Information Extraction (PIE) text-mining web application.

A further formidable challenge will be the reconciliation of literature-based interaction data and HTP data, which are often still in discord. The level of detail and reliability of different studies varies greatly, and has led to a call for semi-quantitative metrics to score interaction reliability. The low affinity protein interactions that often underpin biological network regulation are particularly problematic in this regard, and undoubtedly account for a large fraction of currently uncharted interactions. In addition to focused studies in the literature that often draw on subtle inferences and clever experiments to detect such interactions, the application of new methods, such as protein cross-linking followed by mass spectrometric deconvolution, should help increase the rate of detection of transient regulatory interactions.

The biomedical research community is now beginning to leverage the wealth of network data across multiple species to gain a better understanding of human health and disease [9]. As the deluge of genome sequence data associated with cancer and other diseases continues to mount, a cross-species approach that draws on experimentally tractable model species will be a key step toward understanding the function of conserved but poorly annotated human genes. To provide the necessary underlying data for these efforts, interaction and phenotype curation has been expanded by our group and others to capture data across the major model organism species, including bacteria, viruses, yeasts, plants, nematode, fruit fly, zebrafish, mouse, rat, primate, and human [4]. These curation efforts are often focused on central biological processes or diseases. For example, we have recently undertaken exhaustive curation of the extensive interaction networks that control the state of chromatin modification and protein ubiquitination in both yeast and humans [4]. Similarly, the Gene Ontology Consortium has undertaken initiatives to describe specific developmental and disease-associated processes [6]. A related challenge is the elaboration of generic interaction networks that often lack context specificity towards more realistic dynamic networks that incorporate information on particular cellular contexts, developmental states or disease conditions. This task will require both more detailed annotation and the integration of different data types such as tissue-specific expression and precise phenotypes [13]. To begin to address this, our curation efforts have begun to include the regulation of protein interactions by post-translational modifications, the specific contexts or conditions under which the interaction occurs, and the classification of genetic interactions according to quantitative phenotypes [4].

The utility of the comprehensive yeast interaction dataset that we described in 2006 has grown well beyond our original simple intended application as a benchmark for HTP datasets. BioGRID now houses a vast amount of data from multiple species, and is a general resource for experimental computational biologists alike. The BioGRID, its partner interaction databases, model organism databases, and public meta-resources will all play a crucial role in biomedical research in the post-genomics era. We close this brief overview by noting that there is an urgent need to develop the equivalent of a unified human model organism database that incorporates protein and genetic interaction data, regulatory data at the DNA, RNA and protein levels, polymorphism and disease-associated sequence variation data, quantitative phenotypic data, and drug-target interaction data. These integrated datasets will eventually set the stage for sophisticated computational models able to predict cellular behavior, disease outcomes and new modes of therapeutic intervention.

## Note

This article is part of the *BMC Biology *tenth anniversary series. Other articles in this series can be found at http://www.biomedcentral.com/bmcbiol/series/tenthanniversary.
